# Feasibility and Advantages of Continuous Synthesis of Bioinspired Silica Using CO_2_ as an Acidifying Agent

**DOI:** 10.1021/acssuschemeng.4c03101

**Published:** 2024-06-21

**Authors:** Chinmay A. Shukla, Roja P. Moghadam, Siddharth V. Patwardhan, Vivek V. Ranade

**Affiliations:** † Multiphase Reactors and Process Intensification Group, Bernal Institute, University of Limerick, Limerick V94 T9PX, Ireland; ‡ Green Nanomaterials Research Group, Department of Chemical and Biological Engineering, The University of Sheffield, Mappin Street, Sheffield S1 3JD, U.K.

**Keywords:** sustainable synthesis, pH control, porous particles, surface area, zeta potential

## Abstract

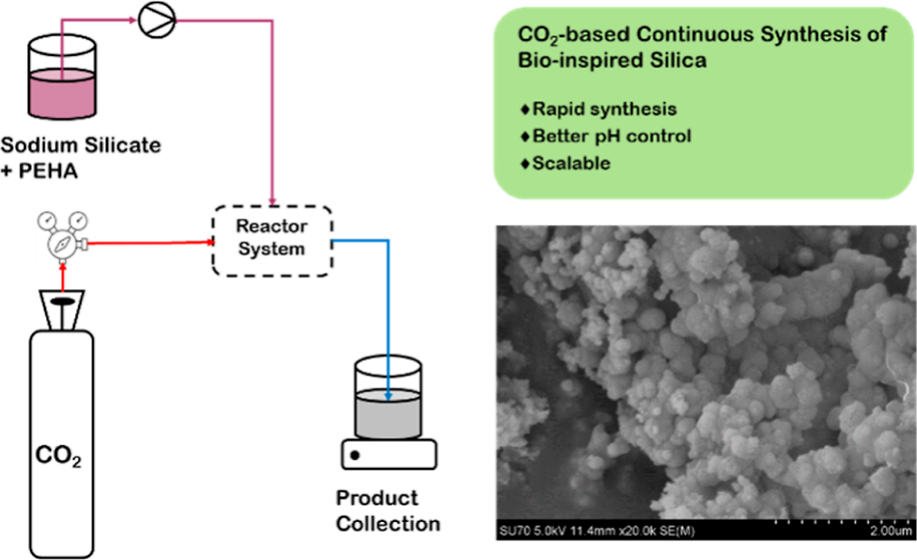

In this work, we present a method for the continuous synthesis of bioinspired porous silica (BIS) particles using carbon dioxide (CO_2_) as an acidifying agent. Typical BIS synthesis uses strong mineral acids (e.g., HCl) to initiate the hydrolysis and subsequent condensation reactions. The use of strong acids leads to challenges in controlling the reaction pH. The synthesis approach proposed in this work offers for the first time CO_2_ as an attractive alternative for the synthesis of BIS and demonstrates the continuous process. The developed method leverages the mild acidic and the self-buffering nature of the CO_2_ combined with additional options for controlling mass transfer rates to facilitate enhanced control of pH, which is crucial for controlling the properties of synthesized BIS. Proof of concept experiments conducted in continuous mode demonstrated a yield of over 70% and a surface area exceeding 500 m^2^/g. These results indicate the successful synthesis of BIS using CO_2_ with properties in the desired range. The enhanced pH control offered by this CO_2_-based process will facilitate the implementation of a sustainable and robust continuous process for BIS synthesis.

## Abstract 2

The CO_2_-based process for synthesizing bioinspired silica ensures superior pH control, higher yield and surface area, and enhanced sustainability.

## 1 Introduction

Porous silica has wide-ranging applications in catalysis, adsorbents, drug delivery, fillers, and additives for food and drug products and biosensors. Porous silicas are normally synthesized using precipitation, pyrolysis, and sol–gel methods, with or without the use of templates.^1,2^ The conventional methods of synthesizing porous silica particles typically involve organic solvents and flammable and toxic reagents and require long reaction times. These methods also require an energy-intensive calcination step to remove templates. Conversely, biosilica is produced naturally in many biological organisms, viz., diatoms, sponges, and plants. Researchers have found that organic biomolecules present in the organisms, viz., proteins, peptides, and polyamines play important roles in the formation of biosilica. Inspired by this, researchers have extracted and isolated the biomolecules and used them for synthesizing silica in vitro.^3−5^ The group of Professor Patwardhan has developed a bioinspired route for synthesizing porous silica particles (hereafter called as bioinspired silica, BIS) which offers fast synthesis at ambient conditions and does not require a calcination step.^6^ Their synthesis method is significantly more sustainable.^7^ In this work, the focus is on further improving the BIS synthesis process reported in these works.

The BIS synthesis uses an aqueous medium and low-cost precursor sodium metasilicate.^8,9^ The synthesis involves acidification of a silica precursor in the presence of a bioinspired additive (such as amines) leading to rapid precipitation of BIS. The synthesis step is rapid and completed in 5 min at room temperature (∼20 °C). The additive can be extracted completely by acid elution at room temperature (∼20 °C). By adjusting the pH at the synthesis and elution steps, the properties of synthesized BIS (primarily surface area and zeta potential) can be tailored so as to generate products of the desired critical quality attributes (CQAs). Optimization studies of the BIS synthesis route indicated that controlling the pH at the synthesis step is the most important process parameter for tailoring the properties of synthesized BIS.^8^ These studies used aqueous hydrochloric acid (HCl) as the acidifying agent. Because of its high dissociation constant and high reactivity, strong acids like HCl cause rapid pH changes which are often controlled by local micromixing. The local pH distribution is therefore determined by complex interactions of the rate of addition of HCl, local mixing at and around the tip of the addition pipe, and reactant concentrations. The pH control in such a semibatch process of BIS synthesis involving strong acid like HCl is therefore quite challenging. In this work, we present an improved BIS synthesis process by using gaseous CO_2_ as an acidifying agent and by developing a continuous process.

CO_2_ is a weak acid. CO_2_ also has a self-buffering nature due to the equilibrium between carbonic acid, bicarbonate, and dissolved CO_2_, which can help stabilize the reaction pH.^10^ Considering this potential for better control of pH, in this work, we developed a gaseous CO_2_-based BIS synthesis process. Furthermore, the use of a gaseous acidifying agent provides us with an additional lever to control the pH at the synthesis step by manipulating gas–liquid mass transfer. We present here a proof-of-concept rapid synthesis of BIS using CO_2_ operated in a continuous mode. The presented approach and results will be useful for further work on modeling, optimization, and scale-up of this novel CO_2_-based BIS synthesis process.

## 2 Experimental Section

### 2.1 Batch and Semibatch Experiments

Initially, batch or semibatch experiments were designed to evaluate the feasibility of using CO_2_ as an acidifying agent for the synthesis of BIS. The main objective was to analyze and assess the properties of BIS synthesized using CO_2_ in comparison with the earlier reported method.^9^ These preliminary experiments were carried out in glass beakers/reactors with magnetic stirrers. Initially, the experiment was carried out with aqueous HCl, which serves as a benchmark when compared with the CO_2_ process. Sodium silicate pentahydrate and pentaethylenehexamine (PEHA) as a template were dissolved in DI water to get final concentration of 30 and 5 mM, respectively. All the required HCl was added in one shot (addition within 2 s), and batch time was measured after completing the HCl addition. The pH was measured using a METTLER TOLEDO SevenExcellence S470 Benchtop Meter. The pH reached 7 for HCl experiments at the end of 5 min (see Figure 2), which is the end point of the reaction. The molar ratio of sodium silicate pentahydrate, PEHA, and hydrochloric acid (HCl) was 1:0.17:2.45 for batch experiments. After the synthesis, 1 M HCl was used to adjust the pH of the reaction mixture to ∼2 to remove PEHA (acid elution) from the BIS particles. The reaction mixture was stirred for 5–10 min after maintaining the pH at 2 and then centrifuged and washed multiple times to remove salt byproducts with DI water until the conductivity of the supernatant was less than 5 μS/cm. The slurry was spread on a Petri dish, and water was evaporated at ambient conditions. The particles were further dried in an oven at 65 °C for further analysis. After this HCl experiment, preliminary experiments with gaseous CO_2_ were carried out in a semibatch mode. Unlike HCl where addition was done in one shot, the CO_2_ gas was bubbled throughout the experiment. The post-synthesis procedure was the same as that used for the HCl experiments.

### 2.2 Continuous Experiments Using HCl and CO_2_ as Acidic Agents

After the establishment that gaseous CO_2_ can be used for producing BIS, continuous synthesis experiments were designed and executed. For comparison purposes, continuous experiments were also carried out with aqueous HCl as an acidifying agent. All experiments were performed at ambient temperature (18.53 ± 1.68 °C), and each experiment was at near the isothermal condition.

**Figure 1:**
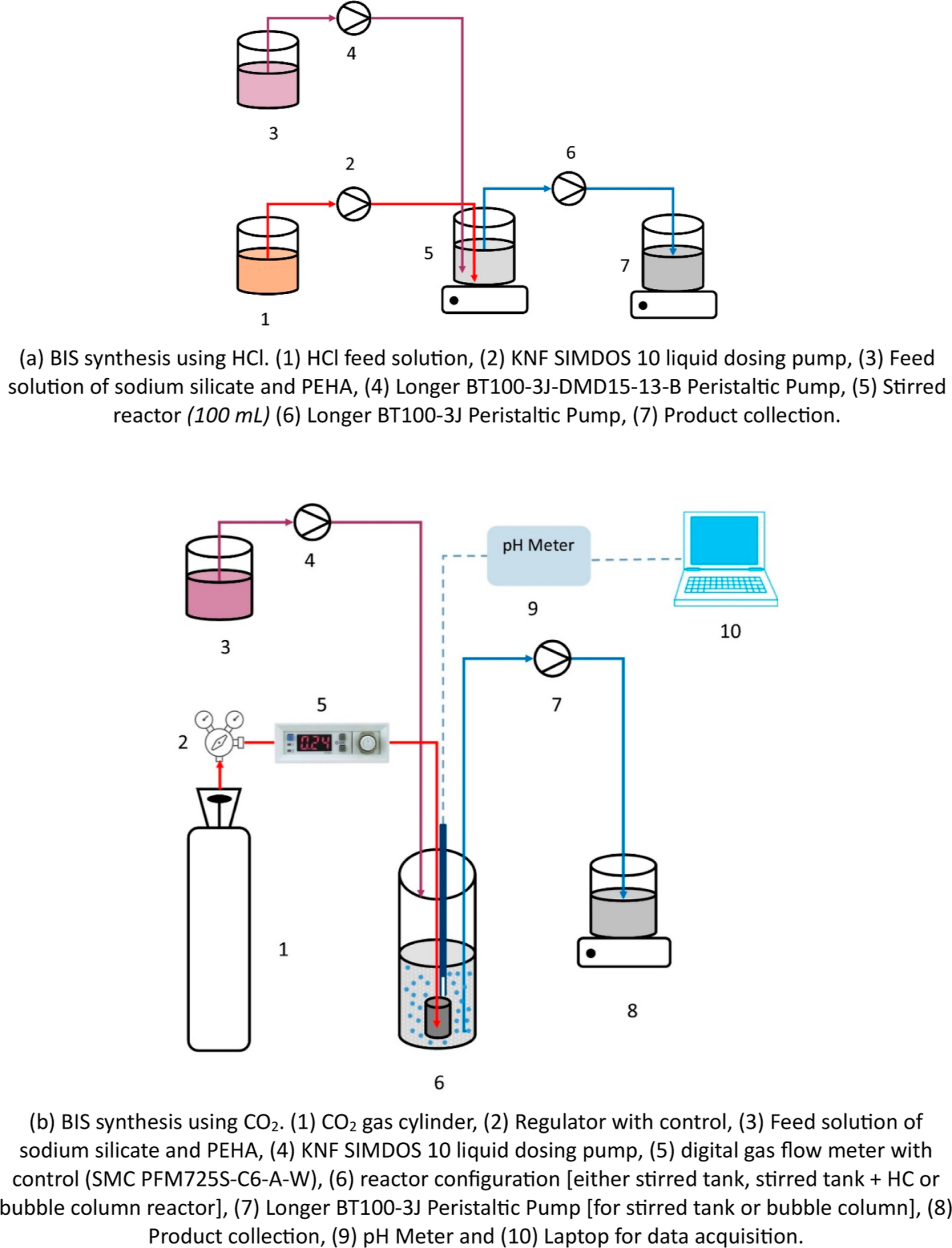
Schematic of experimental setups used with (a) HCl and (b) CO_2_ as the acidifying agents.

The experimental setup used for continuous BIS synthesis using HCl is shown schematically in Figure 1a. The reactor configuration is given later in this discussion (see Configuration A). The stock solutions of sodium silicate pentahydrate (1.0 M), PEHA (0.166 M) and HCl (2.0 M) were prepared in DI water. HCl stock solution (73.6 mL) was dissolved in DI water to get 1 L feed solution with a concentration of 0.15 M (unit 1 in Figure 1a). Further, sodium silicate stock solution (60 mL) and PEHA stock solution (60 mL) were diluted together with DI water to get 1 L feed solution with feed concentrations of 60 and 10 mM, respectively (unit 3 in Figure 1a). Silicate-PEHA feed solution was dosed using a Longer Peristaltic pump (BT100-3J-DMD15-13-B, unit 4 in Figure 1a), and HCl solution was dosed using a KNF SIMDOS 10 liquid dosing pump (unit 2 in Figure 1a). The residence time was 10 min for all experiments. A longer BT100-3J YZ1515x peristaltic pump (unit 6 in Figure 1a) was used for the outflow from the stirred reactor. The mole ratio of HCl was varied from 2.45 to 2.95. The calibration of the pump’s flow rate was conducted using a Mettler Toledo weighing scale to ensure accuracy with both water and silica slurry. Before starting the experiment/reaction, the liquid level in the reactor was monitored by pumping DI water for at least 2 h. The outlet flow rate was periodically measured to ensure a constant flow rate throughout the experiment.

The schematic of the experimental setup used for continuous experiments with CO_2_ is shown in Figure 1b. Photos of experimental setups are shown in Figures S1 and S2 of the Supporting Information. Continuous experiments with CO_2_ were performed at 30 and 60 mM initial concentrations of sodium silicate pentahydrate. The mole ratio of sodium silicate pentahydrate and PEHA was 1:0.17 in all the cases. The feed solution (in unit 3 of Figure 1b) was dosed using KNF SIMDOS 10 liquid dosing and metering pumps (unit 4 in Figure 1b). A CO_2_ cylinder with a manual regulator (units 1 and 2 respectively in Figure 1b) was used to control the gas flow rate. In early experiments, the CO_2_ flow rates were ∼2.3 LPM. The gas flow rate was adjusted to get the desired pH and was always in stoichiometric excess. During long operation times, the CO_2_ pressure from the source cylinder may drop, creating fluctuation in the gas flow rate. This may lead to a slight deviation in the reaction pH. The flow rate was controlled manually to ensure that the flow rate was maintained at the desired values. The following three different reactor configurations were used for carrying out these CO_2_ experiments:

- Configuration A: A stirred tank reactor (Corning Gosselin Straight Container) with a diameter of 52 mm and liquid height of 57 mm (volume = 100 mL) was used. A magnetic stirrer with a stirrer bar diameter of 7 mm and length of 20 mm was operated at 700 rpm. This mixing/RPM in the absence of gas bubbling was sufficient to suspend the solid silica particles. This configuration was also used for continuous HCl experiments.
- Configuration B: A stirred reactor followed by a Helical Coil (HC) (ID = 4 mm and length = 5 m) in series (total volume = 175 mL). The other operating conditions for the stirred reactor were the same as Configuration A.
- Configuration C: A bubble column reactor (diameter = 67 mm and reactor volume = 200 mL) was used.

**Figure 2:**
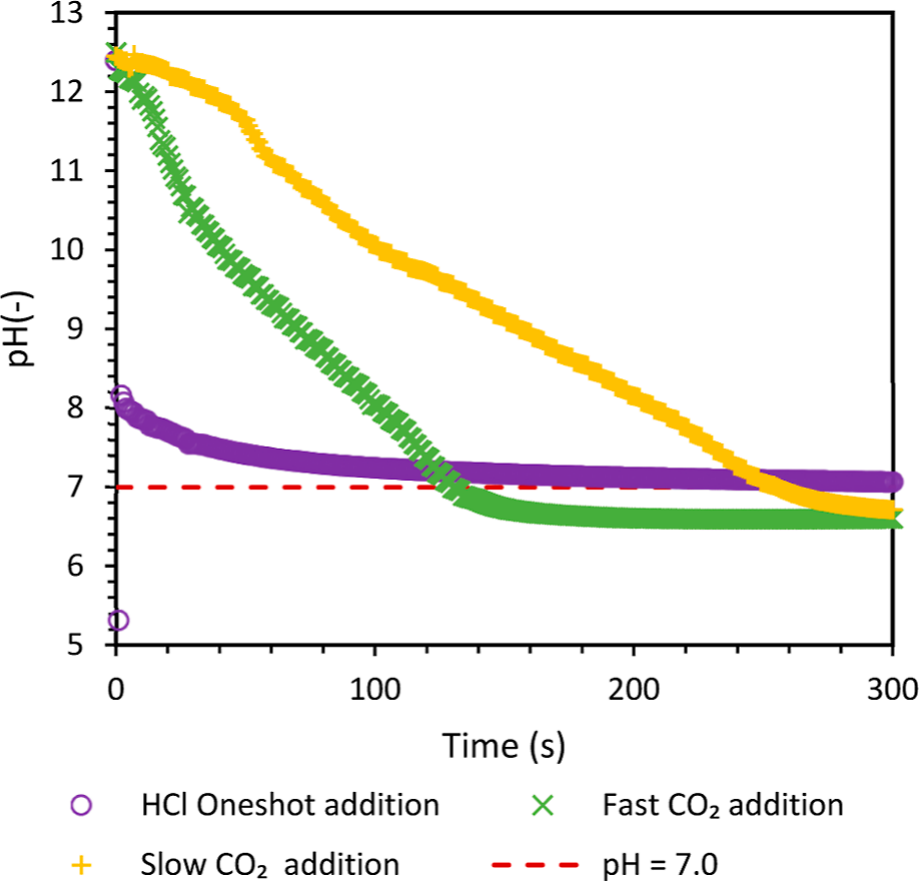
Experimentally observed pH profiles (reactor: 50 mL scale). The mole ratio for HCl was 2.45, while that for CO_2_ was in excess with respect to sodium silicate. The standard deviations in the final experimental pH values were ±0.026 and ±0.042 for HCl and CO_2_ experiments, respectively.

**Figure 3:**
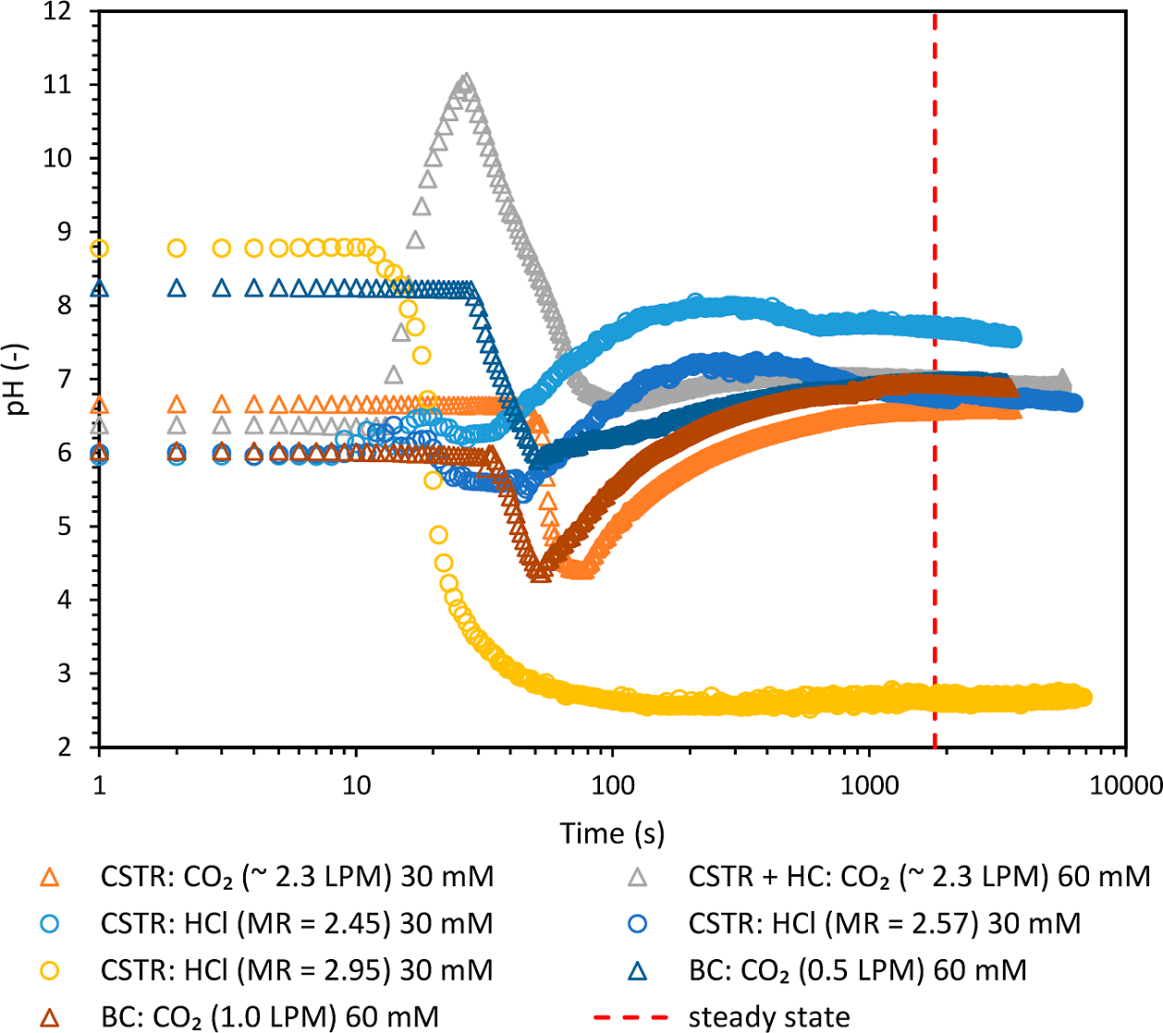
pH profiles obtained with different reactor configurations and different acidification agents. The standard deviations in the steady-state experimental pH values were ±0.0565 and ±0.006 for HCl and CO_2_ experiments, respectively.

The operating parameters for implementing the BIS process with a bubble column were selected based on preliminary estimates of gas hold-up and mass transfer coefficients. Using the correlations of Akita and Yoshida,^11^ the mass transfer time scale (t_MT_) was estimated to be of the order of 10^2^ s. This is at least three (if not more) orders of magnitude larger than the estimated reaction time scales. Therefore, the availability of CO_2_ in the liquid phase can be considered as mass transfer-controlled. Based on the estimated mass transfer time scale, the residence time of 10 min was sufficient. Hence, all the experiments were therefore carried out with the residence time of 10 min. The steady state was observed after 3 residence times (see Figure 3). The samples were collected after four residence times. Two samples were collected separately in a stirred beaker. The sample collection time was 30 or 60 min for each sample. The first sample was directly centrifuged, washed, and dried without any acid treatment (at the reaction pH). The second sample was treated with hydrochloric acid to achieve pH 2 (PEHA removal step) and then centrifuged, washed, and dried as described in the previous section. All experiments were operated at least for 10 residence times. No clogging was observed during the experiment; however, some occasional fouling of the tubes was observed. The potential errors in the pH, conductivity, and temperature were ±0.002, ±0.5%, and ±0.1 °C respectively, as per the manufacturer’s specifications. The pH/conductivity probes were calibrated every time before the experiment with standard solutions to address uncertainty. Furthermore, the KNF dosing pump had an accuracy of ∼±2% of the set-point value.

### 2.3 Silica Particle Characterization

The BIS samples were analyzed to measure surface area, zeta potential, particle size distribution, and morphology. For surface area analysis, Micromeritics TriStar II Plus 3030 nitrogen adsorption equipment was used, and analysis was performed at 77 K. Around 70–80 mg of the dried sample was degassed at 120 °C for ∼17 h. BET analysis with Rouquerol criteria was used for the surface area calculation. Rouquerol criteria are commonly used for measuring microporous particles’ surface area.^12^ Zeta potential was measured using a Zetasizer Nano (Malvern Panalytical). Silica suspension of dried samples was prepared in DI water with a concentration of 0.25 mg/mL by sonication. ∼1 mL aliquot of this suspension was analyzed in a capillary cell, and 10–20 measurements were taken for each sample at 20 °C. Particle size was analyzed using Mastersizer 3000 (Malvern Panalytical). 1 mL of suspended silica slurry was added to the HydroMV unit of Mastersizer, and 3–6 measurements were taken for each sample. Water was used as a dispersant, and 500 rpm was used. It should be noted that the size of primary silica particles is expected to be around 10s of nm (which is not detectable using Mastersizer). These primary particles aggregate into secondary particles of 200–400 nm.^13^ These aggregates further combine to form much larger agglomerates (microns in size) in suspension depending on the pH of the solution or surface charge of silica particles.^14^ The size distribution measured in this work corresponds to the size of such tertiary agglomerates. For convenience, we have termed the agglomerates as “particles”. Dried samples were analyzed using Hitachi SU-70 Scanning Electron Microscopy at 5 kV. Thermogravimetric Analysis was performed by PerkinElmer TGA 4000, from 30 to 400 °C at 20 °C/min and with nitrogen. Around 4–10 mg of the dried sample was utilized for the analysis. More details on material analysis and characterization are given in the Supporting Information (see Figures S3–S15).

## 3 Results and Discussion

**Scheme 1:**
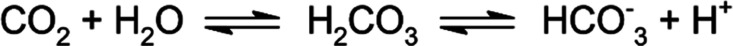
Self-Buffering of Carbonate

Initially, semibatch experiments were carried out to synthesize bioinspired silica using HCl and dry ice as a source of CO_2_. The initial pH for all experiments was the same (∼12.4). As mentioned in the previous section, HCl was added in one shot at the beginning of the batch experiments. The experimentally observed pH profiles using HCl and CO_2_ as acidifying agents are shown in Figure 2. With a one-shot addition of HCl, the recorded pH immediately reduces to 5.3 and quickly rises to more than 8 (within the first 2 s of addition). The pH then gradually decreased to 7 in about 4 min. CO_2_ was added in two modes: fast addition mode (during the initial phase where the CO_2_ release rate from dry ice was higher) and slow addition mode (during the later phase where the CO_2_ release rate from dry ice was lower). With CO_2_, the pH decreases gradually first and then rapidly reaches the desired pH of 7. The gradual decrease in pH is more pronounced during slow CO_2_ addition. It is important to note that CO_2_ was continuously added during the entire experiment, while a predetermined quantity of HCl was added only at the beginning of the experiment. Unlike excess HCl that causes a significant deviation from the desired pH of 7, the continued addition of CO_2_ does not significantly lower the pH, stabilizing around 6.5. This is because of the self-regulation of the pH by CO_2_ and other carbonate ions. CO_2_ reacts with water to form carbonic acid, which further dissociates into bicarbonate ions and hydrogen ions (see Scheme 1). The presence of carbonic acid as a weak acid and carbonate ion as its conjugate base stabilizes the pH. During synthesis, the byproduct sodium carbonate also dissociates into bicarbonate ions. This natural buffer system is leveraged here to achieve enhanced pH control during the synthesis process.^10^

This self-regulated pH is dependent on the concentration of bicarbonate. The bicarbonate is largely formed from the consumption of sodium silicate. Therefore, for a higher concentration of silicate (60 mM), the pH stabilizes at ∼7, whereas for the lower silicate concentration (30 mM), the pH reaches a value of ∼6.5.

The isolated yield with HCl was 60%, which was comparable to the reported yield.^9^ A slightly lower yield (∼50%) was obtained from these preliminary CO_2_ experiments. After verifying the feasibility of using CO_2_ gas for the synthesis in semibatch experiments, further experiments were carried out in a continuous mode.

For initial continuous experiments, the CO_2_ flow rate, controlled manually by a cylinder regulator, was measured as ∼2.3 LPM. No attempt was made to measure it accurately since it is in excess of the requirements for acidification. The experimentally observed pH profiles for different operating conditions and reactor configurations are listed in Figure 3. Configuration A allowed easy disengagement of gas and liquid and facilitated overall operation. The overall yield using the Configuration A was 48%. The same setup was used for carrying out experiments using HCl for comparison purposes. These continuous experiments were performed with HCl mole ratios of 2.45, 2.57, and 2.95, respectively with respect to silicate. Different mole ratios were selected to understand the pH sensitivity with strong acids. The pH of the HCl feed solution was ∼0.9. A slight variation in the mole ratio of HCl resulted in a significant variation in the steady-state pH (Figure 3). Furthermore, a large difference between the p*K*_a_ of HCl (−6.3) and set point pH of 7 makes it challenging to control pH in continuous experiments where a slight deviation/fluctuation in the pump flow rate is detrimental. For better pH control, the difference between the desired pH (7 in the present case) and the p*K*_a_ of the acidifying agent should be minimal.^15^ The examination of p*K*_a_ values of different acids viz. CO_2_ (6.35), acetic acid (4.756), and HCl (−6.3) clearly indicates the superiority of CO_2_, making it the optimal choice for enhanced and precise pH control. This is also evident in Figure 3. The use of acetic acid was not considered here and is only shown for reference, as CO_2_ is a relatively safe and sustainable choice compared to acetic acid.

**Figure 4:**
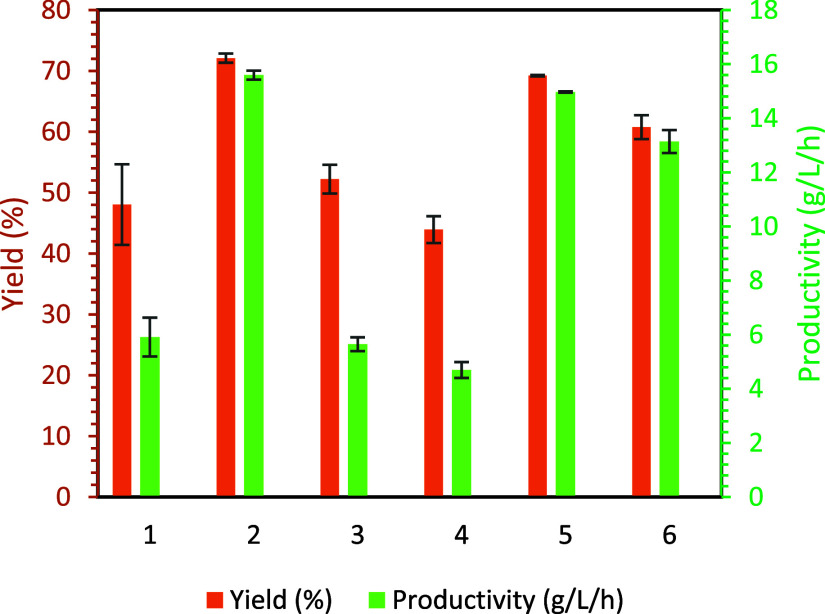
Yield and productivity of silica based on pH = 2 samples for continuous experiments. (1) CSTR with CO_2_ (∼2.3 LPM), (2) CSTR + HC with CO_2_ (∼2.3 LPM), (3) CSTR with HCl (MR = 2.45), (4) CSTR with HCl (MR = 2.57), (5) bubble column with CO_2_ (0.5 LPM), and (6) bubble column with CO_2_ (1.0 LPM). Concentrations for silicate and PEHA were 60 and 10 mM for 2, 5, and 6. For all other experiments, half concentrations were used. Configuration A (labels 1, 3, and 4), Configuration B (label 2), and Configuration C (labels 5 and 6).

Significant variations are observed in the initial part (up to 100 s) of the profiles, as shown in Figure 3. Initially, the reactor contained deionized (DI) water, with a pH range of 6–6.7, attributed to dissolved CO_2_ from the atmosphere. The reagent pumps, viz., silicate-PEHA and HCl (in the case of Figure 1a) or the silicate-PEHA pump and CO_2_ cylinder (in the case of Figure 1b) were started shortly after, introducing a practical challenge in achieving simultaneous initiation of both pumps or pump and cylinder. This resulted in a time lag of a few seconds, influencing the initial pH trend. Specifically, the order of entry of silicate-PEHA (basic) and HCl or CO_2_ (acidic) impacted the pH trajectory in the initial part. For instance, when silicate entered first, the pH increased up to 11 before stabilizing (Δ gray triangle data in Figure 3). Conversely, if acid (HCl or CO_2_) entered first, then the pH initially decreased before stabilizing. In one experiment (○ yellow circle data), accidental silicate drops inside the reactor led to an initial pH of ∼8.7. Importantly, the transient pH profiles observed during this start-up process did not influence the product yield or properties, as product collection occurred only after achieving a steady-state reactor pH. The mole ratio of 2.95 resulted in the lowest pH of 2.5 among all experiments, and it did not result in any precipitation of silica particles. In the case of CO_2_ experiments, the lowest pH was 6.5, even though the CO_2_ was in large excess. This can be attributed to the mildly acidic, limited solubility, and self-buffering nature of CO_2_ as discussed earlier. To understand the influence of silicate concentration, an experiment was performed by doubling the silicate and additive concentration (60 and 10 mM, respectively) in Configuration B (a CSTR and helical coil in series). This experiment resulted in an improved yield of 72 ± 0.76% and throughput of 15.6 ± 0.16 g/h/L. The yield obtained on the larger scale Configuration C was also ∼70%. The obtained isolated yield and reactor productivity results are shown in Figure 4.

**Figure 5:**
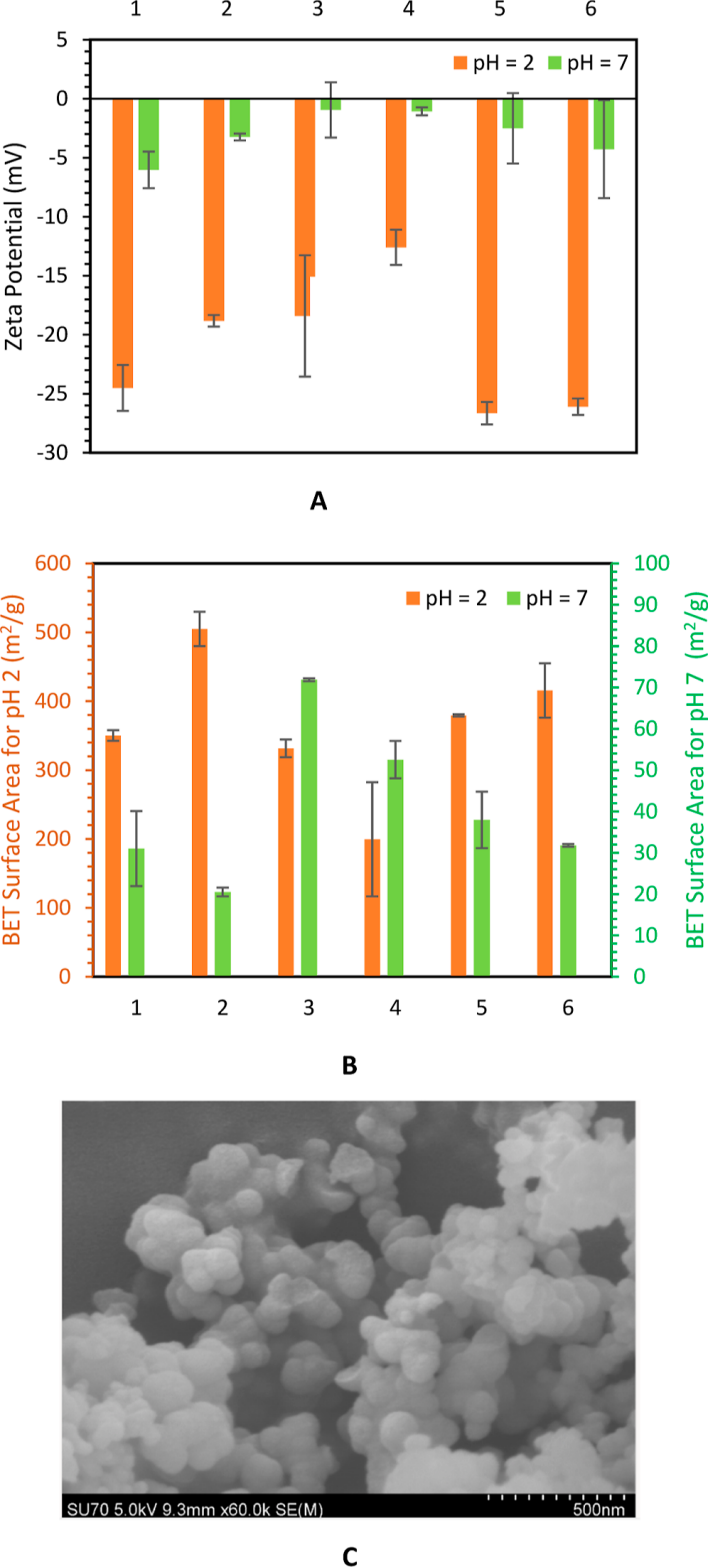
Silica particle characterization for the continuous experiments. (A) Zeta potential, (B) total surface area obtained from BET analysis, and (C) SEM image for a bubble column reactor with CO_2_ (0.5 LPM) silica samples (pH 7). Legend label, (1) CSTR with CO_2_ (∼2.3 LPM), (2) CSTR + HC with CO_2_ (∼2.3 LPM), (3) CSTR with HCl (MR = 2.45), (4) CSTR with HCl (MR = 2.57), (5) bubble column with CO_2_ (0.5 LPM), and (6) bubble column with CO_2_ (1.0 LPM). Concentrations for silicate and PEHA were 60 and 10 mM for 2, 5, and 6. For all other experiments, half concentrations were used. Configuration A (labels 1, 3, and 4), Configuration B (label 2), and Configuration C (labels 5 and 6).

The produced silica particles were then characterized by measuring zeta potential, surface area, particle size distribution, and SEM. BIS has shown potential use as drug delivery systems with the surface area and zeta potential as important critical quality attributes.^16^ The results of zeta potential and surface area of the synthesized BIS particles are shown in Figure 5a,b, respectively. The values of zeta potential are comparable with the reported BIS values.^9^ The pH 2 silica samples have a relatively higher magnitude of zeta potential compared to pH 7 samples due to the absence of PEHA in the silica particles. Since the desired synthesis pH is 7, the “pH 7” sample is symbolic and corresponds to the actual pH at synthesized conditions. The obtained values of surface areas are slightly higher than typical surface areas for bioinspired silica using HCl.^9^ As expected, the total surface areas for pH 2 samples are significantly higher than those for the pH 7 samples, as PEHA is removed from the silica pores during the acid elution step. More details about pore size distribution and particle size distribution are given in Figures S4 and S6–S11. The zeta potential, surface area, and SEM images of BIS synthesized with the bubble column reactor are shown in Figure 5. The zeta potential (−26 mV for pH 2 samples and −3 for pH 7 samples) is comparable for both the CO_2_ flow rates considered in this work. The surface areas for pH 2 samples for 0.5 and 1 LPM experiments were 380 and 415 m^2^/g, respectively. As discussed earlier, the surface area of BIS depends on the synthesis pH and acid elution pH and was not found to be unduly sensitive to the CO_2_ flow rate. The SEM image of the BIS synthesized using CO_2_ in a bubble column reactor (Configuration C) is shown in Figure 5c. Secondary particles from SEM images were analyzed using ImageJ software. More details about the particle size distribution obtained by secondary particles are given in Figure S15. It can be seen that the CO_2_-based process for BIS particles provides much better control of pH and therefore the product properties.

Table 1 shows a comparison of synthesis conditions and silica particle characteristics with CO_2_ as one of the reagents. The majority of the reported processes have batch operation and/or long reaction times, high temperature, and high-pressure operating conditions.

**Table 1:** Summary of Synthesis Conditions (with CO_2_ as One of the Reagents) and Silica Properties Reported in the Literature

synthesis conditions (reactor type, operating conditions, and reagents)	reported silica characterization data	reference
high pressure batch reactor, *T* = 60–80 °C, *P* = 2 bar, reaction time = 1–3 h, aging condition = 50 °C and 2 h	particle size = 15–54 nm, yield = 75.2–88.6% BET surface area = 175 m^2^/g	(17)
sodium silicate, polyethylene glycol 6000 (PEG 6000), CO_2_, and ethanol		
high pressure batch reactor, *T* = ambient temperature, *P* = 0–3 bar, reaction time = 24 h, aging condition = ambient temperature and 2 days	three different particles were synthesized with varying particle size and BET surface areas viz. precipitated silica adsorbent (200–1000 nm and 73.4 m^2^/g), silica gel adsorbent (10–20 nm, 205.9 m^2^/g) and condense silica gel adsorbent (12.0 m^2^/g)	(18)
3-aminopropyltriethoxysilane, sodium silicate and CO_2_		
batch reactor geometry similar to an annular centrifugal extractor, *T* = ambient temperature, *P* = 1 bar, reaction time = 1–5 h	not reported	(19)
sodium silicate, polyethylene glycol as surfactant, ethanol, and CO_2_		
continuous tubular reactor, *T* = 0–80 °C, residence time = 3–120 s	particle size = 150–300 nm	(20)
sodium silicate, ethanol, and CO_2_ or HCl gas		
continuous tubular reactor, *T* = 0–90 °C	BET surface area = 500–800 m^2^/g	(21)
sodium silicate, ammonium salt, and CO_2_		
CSTR or bubble column reactor, *T* = ambient temperature, residence time = 10 min, *P* = 1 bar	yield = 48–72%	the CO_2_-based process—this work
sodium silicate, PEHA, and CO_2_	particle size = 130–188 nm	
	BET surface area	
	pH 2 = 350–505 m^2^/g	
	pH 7 = 20–38 m^2^/g	
	zeta potential	
	pH 2 = −18.8 to −26.6 mV	
	pH 7 = −2.5 to −6.0 mV	

Bioinspired silica is generally spherical in shape; however, they are randomly arranged, making it a disordered type of silica. The synthesis proceeds by the condensation of silicate monomers producing oligomers, which further grow into solid polymeric silica and precipitate from the reaction mixture. The amine additive(s) (PEHA in this case) acts as a catalyst, template, or structure-directing agent. The particles formed during BIS synthesis are classified as primary, secondary, and tertiary particles. Primary particles are typically the main building blocks with sizes ranging from 5 to 10 nm. The secondary particles are obtained by aggregation of these primary particles with typical sizes ranging from 200 to 400 nm. The aggregation is due to chemical interlinking facilitated by amine additives. Hence, aggregation is an inherent nature of BIS synthesis; however, different amine templates can be used to tailor the properties, particularly surface area and zeta potential, to suit the target applications, viz., drug delivery, catalysis, adsorption, battery anodes, tires, etc.^13^

Tertiary particles form due to the agglomeration of these secondary particles (no chemical interlinking). Bioinspired silica particles generally tend to agglomerate during downstream processes, such as centrifugation and drying. Sonication or milling can be employed as a mitigating strategy to break agglomerates. Additionally, surfactants or surface-modifying agents during the synthesis can be used to minimize agglomeration. Other factors such as silica concentration, pH, and zeta potential may also affect agglomeration. Generally, monodisperse silica is important for some applications, viz., photonics, biosensing, and biomedicine.^22^ Because of the agglomeration tendency of BIS particles, its application in these areas may have some limitations. However, despite aggregation and agglomeration, the BIS particles have higher surface areas and are suitable for many applications in catalysis, drug delivery, and adsorption.

Some comments on the scalability of the proposed CO_2_-based BIS synthesis reactor are appropriate here. The BIS synthesis presented here is a typical gas–liquid–solid reaction, with the reaction between the gas (CO_2_) and liquid (containing dissolved silicate and PEHA) phases and the solid as the precipitation product (silica). The reactor design and scale-up criteria will depend on the mixing in the reactor and the gas–liquid mass transfer performance. The typical BIS particles form agglomerates with *d*_43_ in the range of 10–12 μm. The particle settling velocity for such small particles is very low ∼0.35 mm/s, and solid suspension should not pose a significant challenge at a larger scale. Controlling pH in the reactor is critical, which determines yield and particle properties. The pH prevailing in the reactor is primarily determined by gas–liquid mass transfer and mixing in the reactor. The mass transfer time scale of ∼10^2^ s was found to be adequate in the lab scale. Considering that bubble size and volume fraction can be maintained during the scale-up, it is possible to maintain mass transfer performance in the scaled-up reactor similar to the lab scale.^23−25^ A low-aspect ratio bubble column reactor is recommended to facilitate good mixing. Controlling the pH of the reactor and subsequent elution step is critical for achieving the desired performance and will be a key challenge for scaling up. Another important issue in scaling up the BIS process is handling large amounts of silica particles and appropriate management of resulting issues like fouling and clogging.^26,27^ Two key design parameters, namely, (a) prevailing mean velocity and turbulence intensity and (b) material of construction of reactor influence fouling and clogging. Appropriate superficial gas velocity must be maintained to ensure adequate liquid velocity and turbulence intensity to avoid fouling. The choice of a sparger design is critical for avoiding difficulties due to fouling and clogging. The sparger holes are recommended to point downward to avoid clogging. The gas velocity at the sparger holes also needs to be designed to avoid fouling. PTFE-lined reactors, vessels, and pipelines can significantly reduce fouling.^26^ Appropriate choice of material of construction is important considering the transition from an alkaline sodium silicate precursor to a carbonate byproduct and final acidic conditions. PTFE-lined equipment can handle the relevant pH range encountered in the presented process.

Overall, the developed process looks promising, since it allows for better control of pH and thereby improved particle properties. The developed approach allowed for continuous operation for more than 15 residence times without any clogging. The presented results provide a proof of concept for using gaseous CO_2_ for producing BIS and provide a useful basis for reactor design and further work on optimization and process modeling. We hope that this research stimulates further research in this promising area.

## 4 Conclusions

In this study, we developed for the first time a continuous synthesis of BIS using gaseous CO_2_ as an acidifying agent. Our method demonstrates rapid synthesis under mild conditions and offers improved pH control, especially in continuous synthesis. The improved pH control can be attributed to the synergetic effect of mass transfer control, mild acidic nature, and self-buffering nature of the CO_2_. Proof of concept experiments were performed in different reactor configurations. The produced BIS particles were characterized in terms of surface area, zeta potential, and particle size. The surface areas of silica particles up to 505 m^2^/g were produced using CO_2_. The zeta potential ranged from −18.8 to −26.6 mV for pH 2 samples and from −2.5 to −6.0 mV for pH 7 samples. Most of the properties of the synthesized silica are comparable to those reported for BIS synthesized with HCl as acid, with the surface area on the higher side. Both the reactor configurations (stirred tank with a HC and bubble column reactor) gave ∼70% yield and productivity of more than 15 g/h/L. The presented approach and results will be useful for further work toward developing sustainable and scalable process for synthesizing BIS particles of desired characteristics.

## Supporting Information Available

Experimental setup photos, adsorption–desorption isotherm, pore size distribution, Mastersizer RPM optimization, particle size analysis, Zetasizer concentration optimization, TGA analysis, and SEM analysis (PDF)
