# Supplementary material for: Feasibility and Advantages of Continuous Synthesis of Bioinspired Silica Using CO2 as an Acidifying Agent
Source: ACS Sustain Chem Eng. 2024 Jun 21;12(27):10260–8. doi: 10.1021/acssuschemeng.4c03101 (PMC11234356; doi:10.1021/acssuschemeng.4c03101)
Supplement: Supplementary file 1 — sc4c03101_si_001.pdf [file sc4c03101_si_001.pdf]

Feasibility and Advantages of Continuous Synthesis of Bioinspired Silica using CO_2 as an acidifying agent

Chinmay A. Shukla[a], Roja P. Moghadam[a], Siddharth V Patwardhan[b] and Vivek V. Ranade[a]*

[a]Multiphase Reactors and Process Intensification Group
Bernal Institute, University of Limerick, Ireland

[b] Green Nanomaterials Research Group, Department of Chemical and Biological Engineering, The University of Sheffield, Mappin Street, Sheffield, S1 3JD, UK

* Corresponding author: Vivek.Ranade@ul.ie

Supporting Information

Contents

1. Experimental Setup Photos S2
2. Adsorption-Desorption Isotherm S3
3. Pore Size Distribution S4
4. Mastersizer RPM Optimization S4
5. Particle Size Analysis S5
6. Zetasizer Concentration Optimization S8
7. TGA Analysis S9
8. SEM Analysis S10

1. Experimental Setup Photos

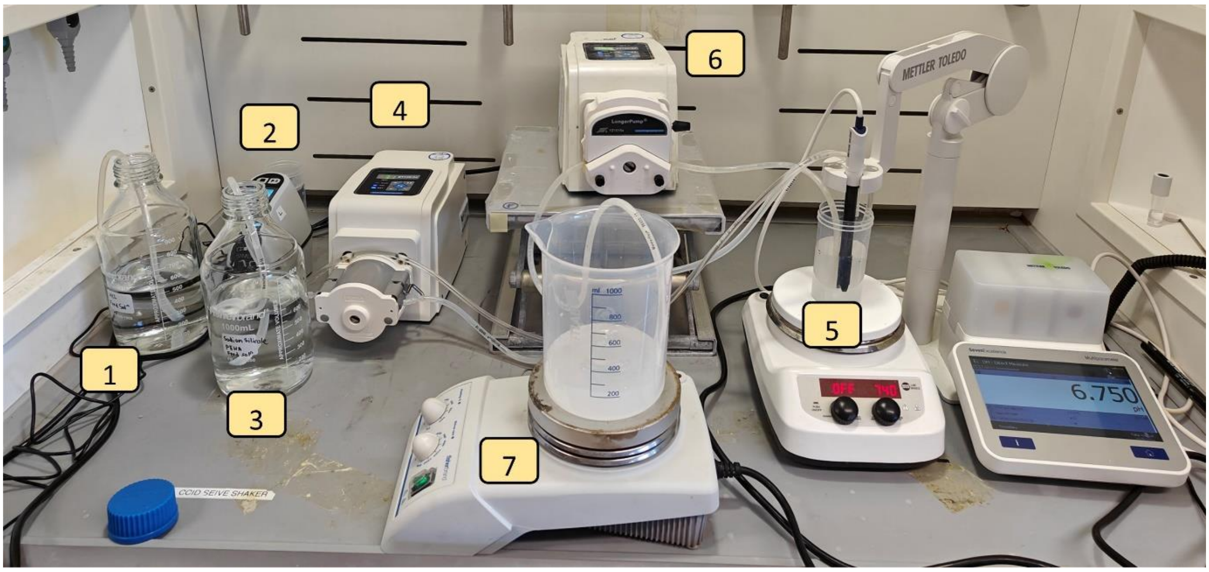

Figure S1. Experimental setup photo corresponding to Figure 1a. Continuous Experimental setup for CSTR using HCl. (1) HCl feed solution, (2) KNF SIMDOS 10 liquid dosing pump, (3) Feed solution of sodium silicate and PEHA, (4) Longer BT100-3J-DMD15-13-B Peristaltic Pump, (5) CSTR (100 mL) (6) Longer BT100-3J Peristaltic Pump, (7) Product collection.

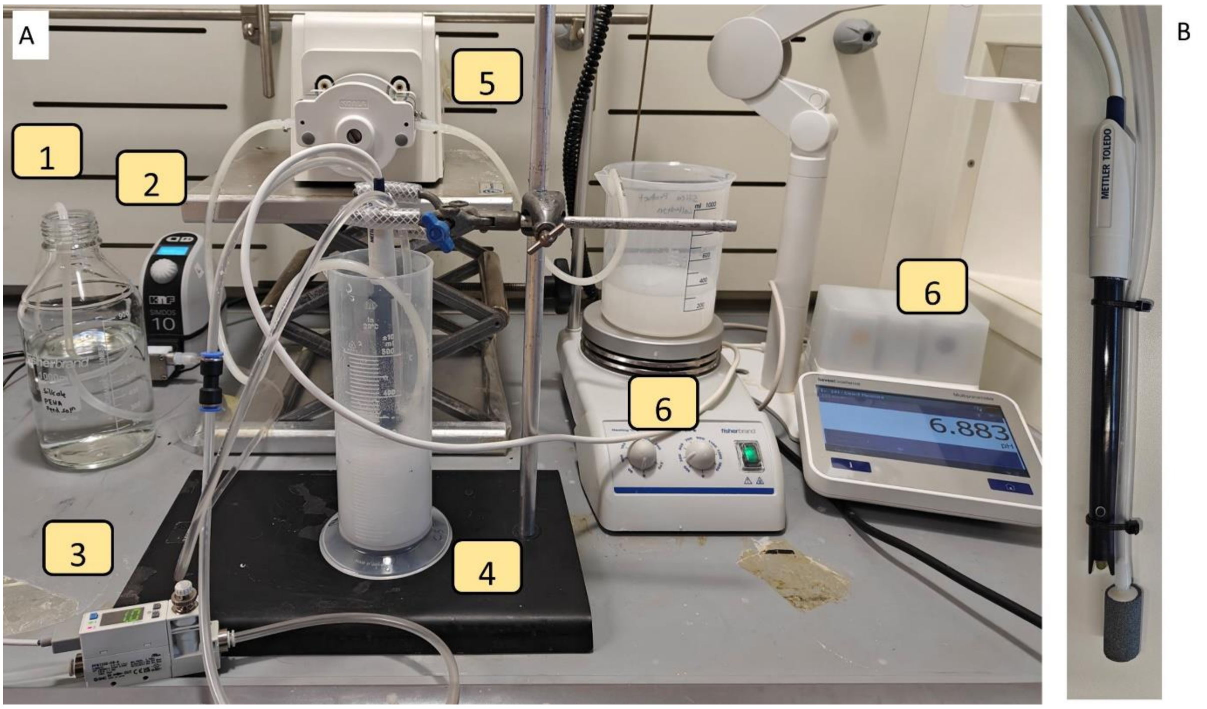

Figure S2. Experimental setup photo corresponding to Figure 1b. A: continuous bubble column setup (1) Feed solution of sodium silicate and PEHA, (2) KNF SIMDOS 10 liquid dosing pump, (3) digital gas flow meter with control (SMC PFM725S-C6-A-W), (4) Bubble column reactor with sparger and pH probe. (5) Longer BT100-3J YZ1515x Peristaltic Pump (6) Product collection, (7) pH meter, and (8) Laptop for data acquisition and B: Assembly of sparger and pH probe. pH probe is used as a support to the sparger gas inlet pipe.

2. Adsorption-Desorption Isotherm

Micromeritics® TriStar II Plus 3030 nitrogen adsorption equipment was used to get adsorption and desorption isotherm, and the experiment was performed at 77 K. Around 70-80 mg dried sample was degassed at 120 °C for ~17 h. More points were acquired at low pressures during adsorption as silica is microporous.

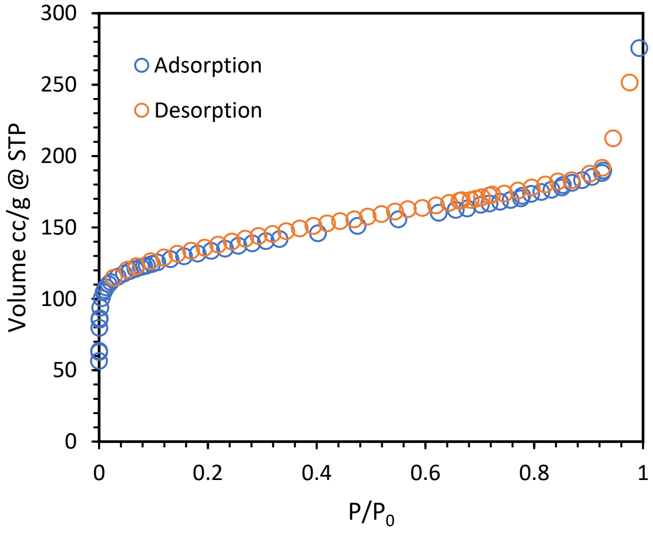

Figure S3. Adsorption-desorption isotherm for CSTR and helical coil in series

3. Pore Size Distribution

Pore size was estimated using the BJH method. Figure S4 shows the pore size distribution of the continuous experiments.

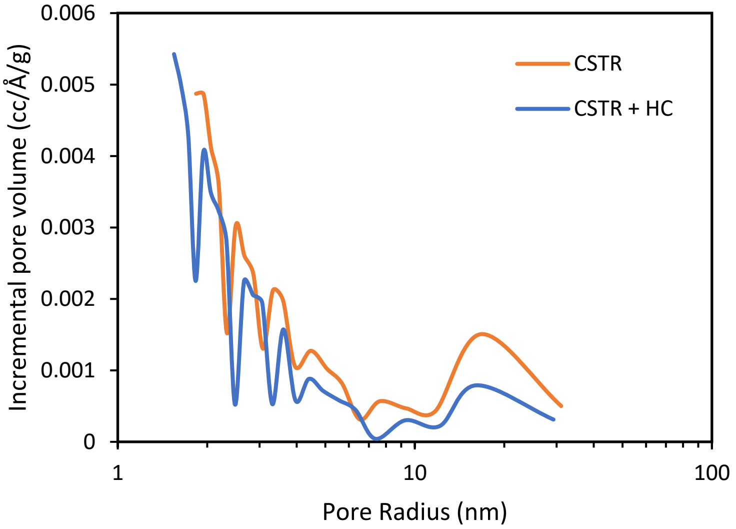

Figure S4. Pore size distribution for continuous experiments

4. Mastersizer RPM Optimization

Particle size was analyzed using Mastersizer 3000 (Malvern Panalytical). 1 mL of suspended silica slurry was added to HydroMV unit of Mastersizer and 3-6 measurements were taken. Water was used as a dispersant and 500 RPM was selected as higher RPM can create air bubble interference [1]. For RPM 500 and 1000 comparable results were obtained.

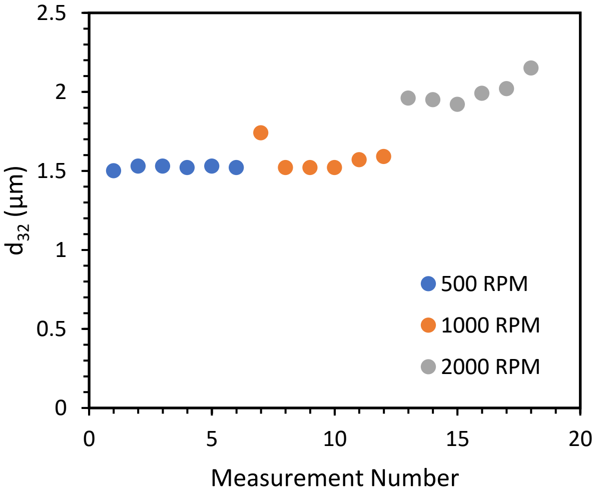

Figure S5. Sauter mean diameter, d_{32} (µm) measured for different RPM.

5. Particle Size Analysis

Figure S6 & S7 shows the particle sizes corresponding to different diameters with HCl and CO_2 as acid for batch & continuous experiments respectively. Figure S8 and S9 shows particle sizes corresponding to different diameters for continuous experiments. Figure S10 and S11 shows the number size distribution from batch experiments and continuous experiments respectively.

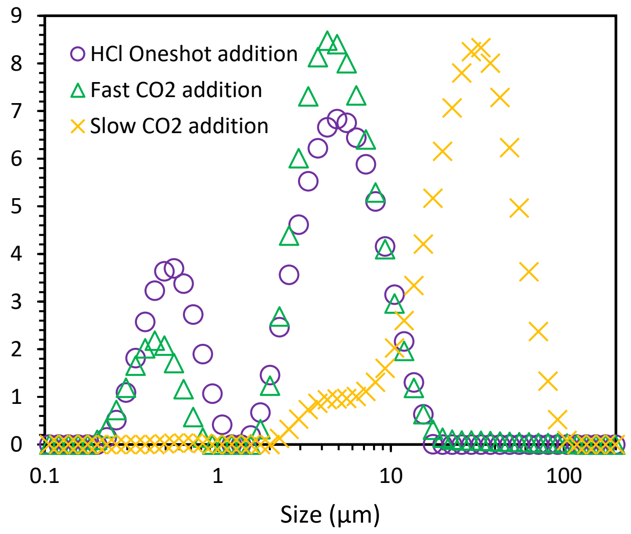

Figure S6. Comparison of particle size distribution for HCl and CO_2 (fast & slow addition). The silica samples after acid treatment (pH = 2) and subsequent washing and drying.

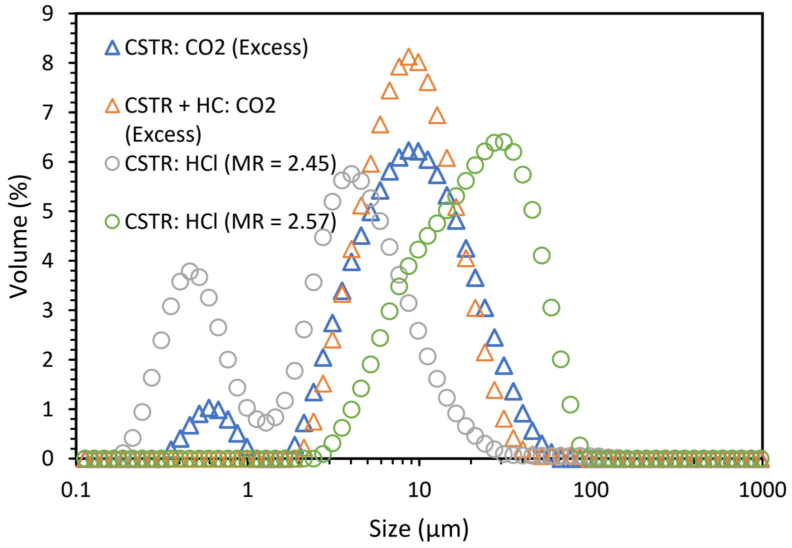

Figure S7. Comparison of particle size distribution for HCl and CO_2 for continuous experiment. The silica samples after acid treatment (pH = 2) and subsequent washing and drying.

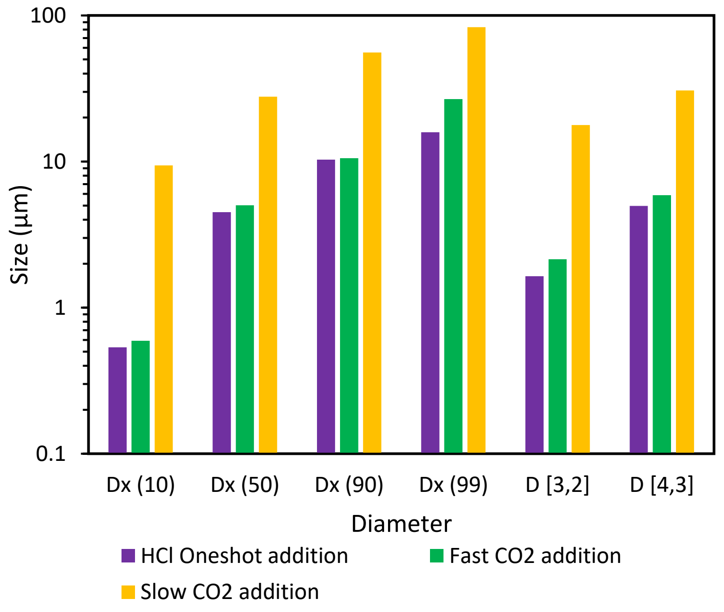

Figure S8. Particle diameters for batch experiments

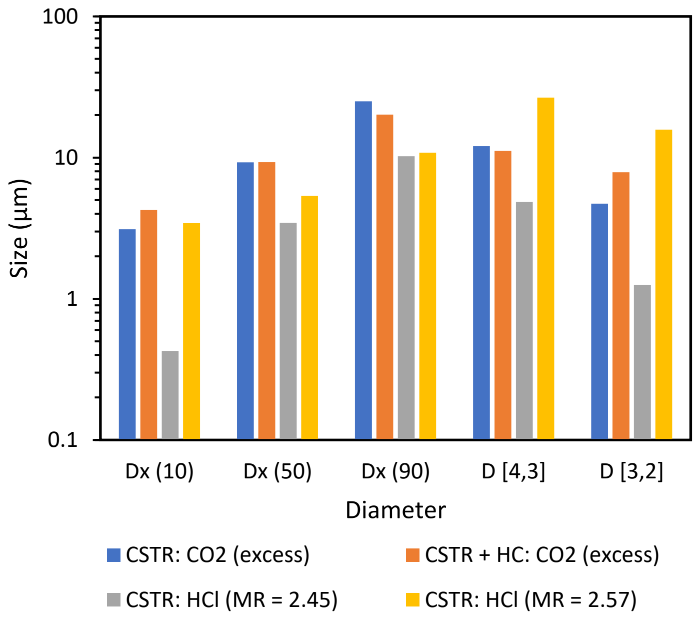

Figure S9. Particle diameters for continuous experiments

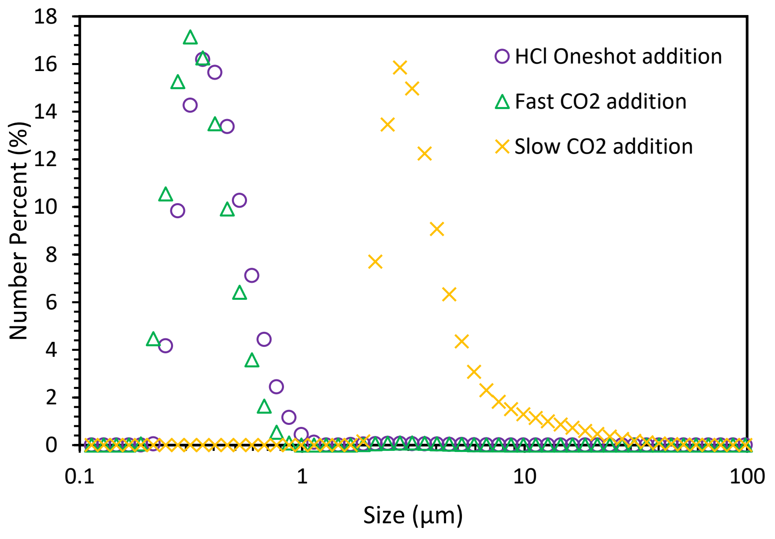

Figure S10. Number size distribution for batch/semi-batch experiments

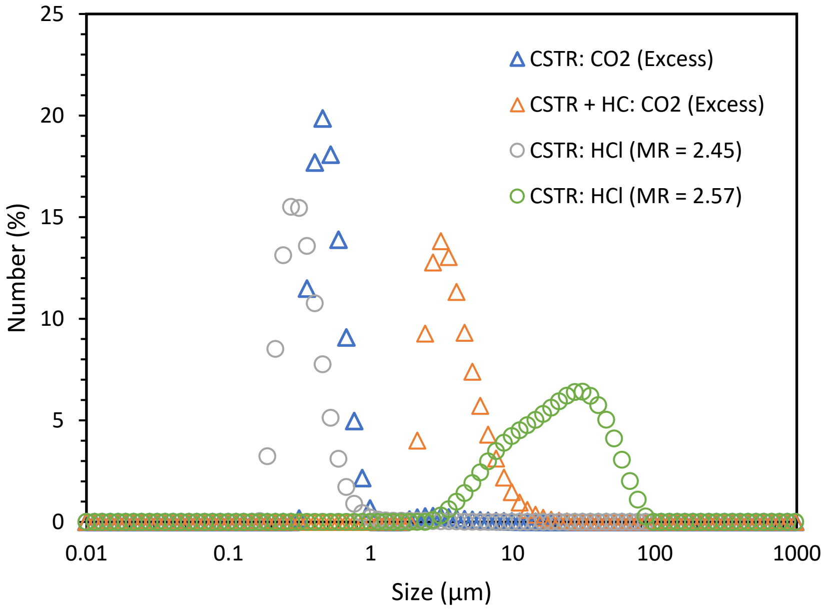

Figure S11. Number size distribution for continuous experiments

6. Zetasizer Concentration Optimization

Zeta potential measurement for synthesised silica sample were measured with different concentrations with three repetitions. According to Figure S12, to concentrations 0.25 mg/mL - 2.5 mg/mL demonstrated comparable results with lower standard deviation. Hence lowest concentration of 0.25 mg/mL was selected for further analysis. Furthermore, higher concentrations have solid settling issues hence was avoided.

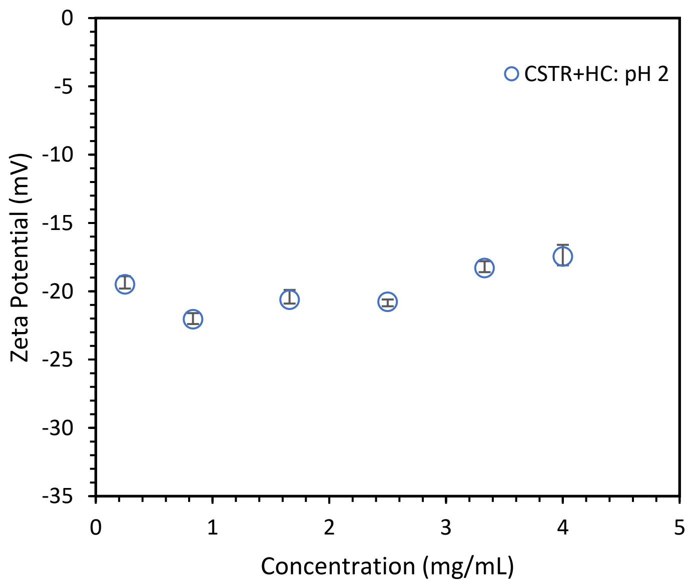

Figure S12. Zeta potential measurement for different concentrations of synthesised silica

7. TGA Analysis

TGA analysis was used to measure the weight % decrease of silica due to moisture and possible PEHA (particularly in pH = 7 samples). The weight % data was used for correcting the isolated yield.

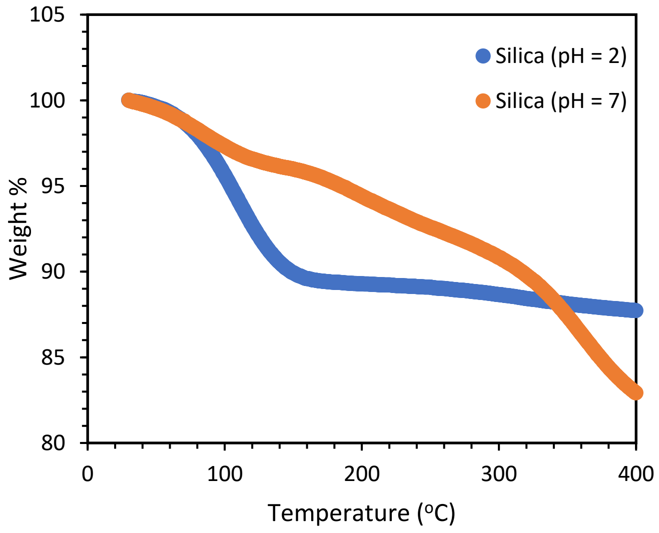

Figure S13. TGA analysis of synthesised silica samples

8. SEM Analysis

SEM images of continuous experiments of CO_2 and HCl experiments with CSTR are given below in Figure S14. The particle shape appears spherical in both cases with presence of aggregates. Figure S15 shows the actual particle size distribution for both the cases. The mean particle size is in the range of 130-188 nm.

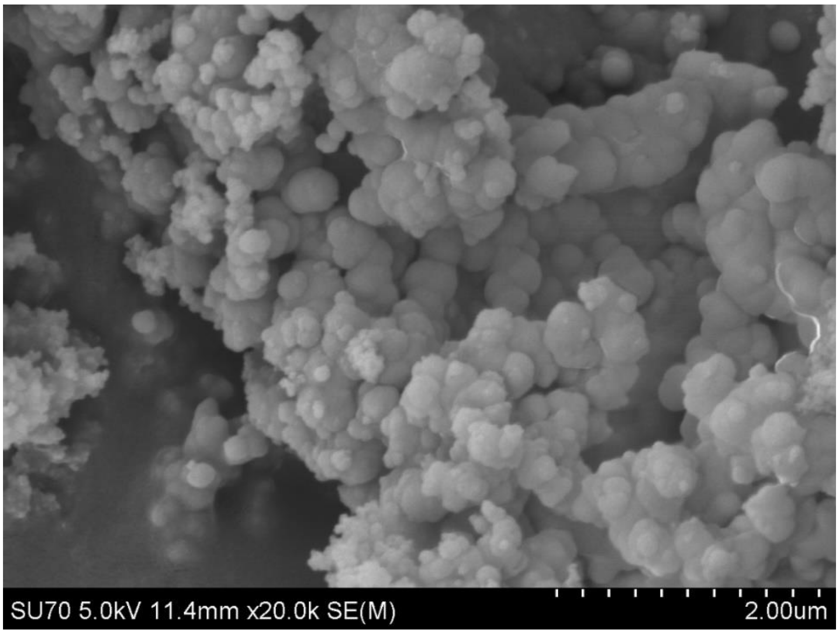

A

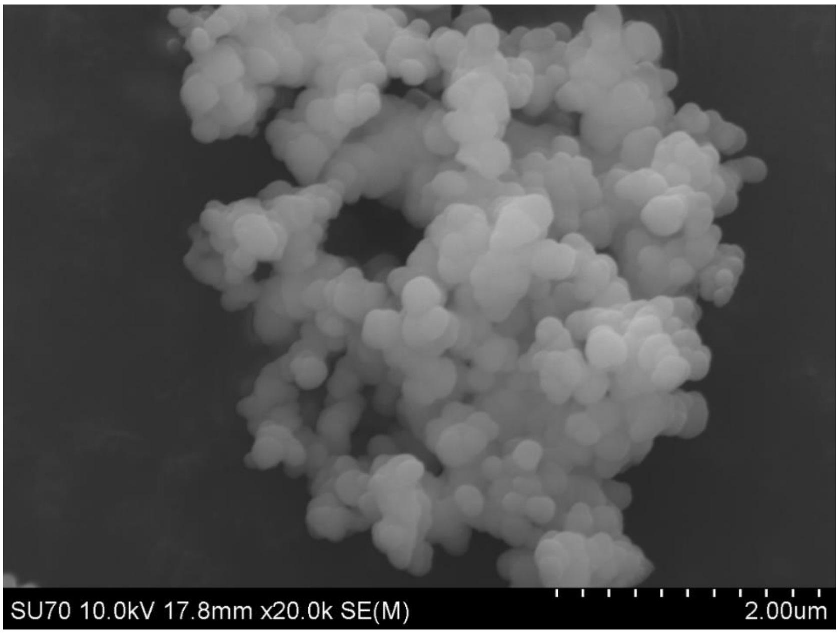

B

Figure S14. SEM images of CO_2 and HCl process. A. CSTR with CO_2 (excess) and B: CSTR with HCl (MR = 2.45)

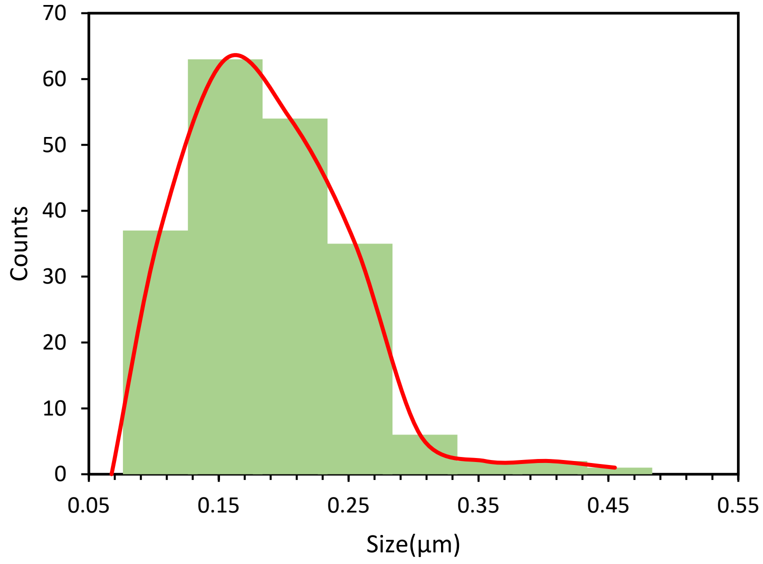

A

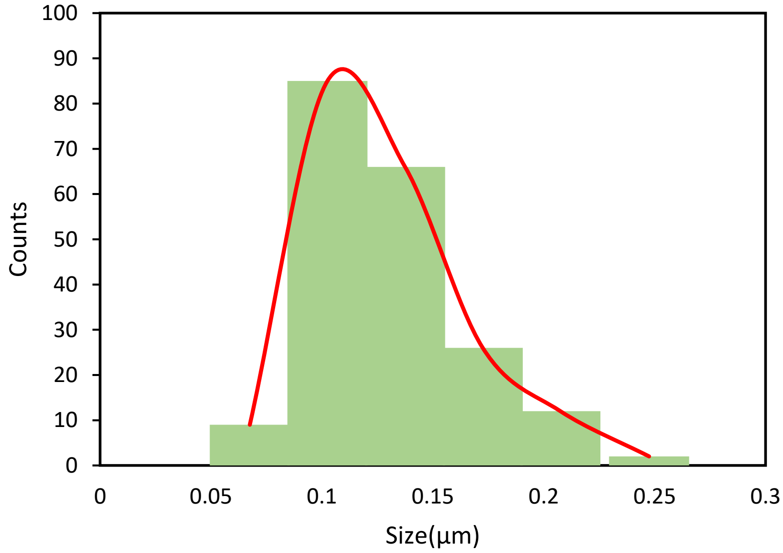

B

Figure S15. Secondary particle size distribution of continuous CO_2 and HCl process. A. CSTR with CO_2 (excess) and B: CSTR with HCl (MR = 2.45)
